# Impact of Obesity and Being Overweight on the Immunogenicity to Live Attenuated Hepatitis A Vaccine in Children and Young Adults

**DOI:** 10.3390/vaccines9020130

**Published:** 2021-02-06

**Authors:** Termpong Dumrisilp, Jongkonnee Wongpiyabovorn, Supranee Buranapraditkun, Chomchanat Tubjaroen, Nataruks Chaijitraruch, Sittichoke Prachuapthunyachart, Palittiya Sintusek, Voranush Chongsrisawat

**Affiliations:** 1 Division of Gastroenterology and Hepatology, Department of Pediatrics, Faculty of Medicine, Chulalongkorn University and King Chulalongkorn Memorial Hospital, Bangkok 10330, Thailand; jusmax_63@hotmail.com (T.D.); chomchanat.t@chulahospital.org (C.T.); Suttiruk.J@chula.ac.th (N.C.); Sittichoke.P@chula.ac.th (S.P.); Palittiya.S@chula.ac.th (P.S.); 2 Division of Immunology, Department of Microbiology, Faculty of Medicine, Chulalongkorn University and King Chulalongkorn Memorial Hospital, Bangkok 10330, Thailand; jongkonnee.w@chula.ac.th; 3 Allergy and Clinical Immunology Unit, Department of Medicine, Faculty of Medicine, Chulalongkorn University and King Chulalongkorn Memorial Hospital, Bangkok 10330, Thailand; bsuprane@chula.ac.th

**Keywords:** hepatitis A, obesity, overweight, immunogenicity, vaccine

## Abstract

Prior results investigating a correlation between obesity and hepatitis A virus (HAV) vaccine response have been inconclusive, with limited data involving live attenuated HAV vaccines. The aim of this study is to evaluate the effect of overweight and obesity on the response to live attenuated HAV vaccine in children and young adults. This prospective cohort study was conducted in Thailand with subjects ranging in age from seven to twenty-five years. The subjects were administered 0.5 mL of MEVAC™-A and tested for anti-HAV antibodies before and at 8–9 weeks after vaccination. Baseline seronegative subjects (anti-HAV antibodies < 20 mIU/mL) were divided into non-obese (underweight/normal weight) and obese (overweight/obesity/severe obesity) groups. A total of 212 (117 non-obese and 95 obese) subjects completed the study (mean age (SD) = 13.95 (3.90) years). The seroprotection rates were 100%. Postvaccination geometric mean titers (95% CI) were 429.51 (401.97, 458.94) and 467.45 (424.47, 514.79) mIU/mL in the non-obese and obese groups, respectively. Females (*p* = 0.013) and subjects with truncal obesity (*p* = 0.002) had significantly higher titers than other participants. Live attenuated HAV vaccine is safe and has comparably high immunogenicity in both underweight/normal weight and overweight/obese persons.

## 1. Introduction

Childhood obesity is one of the most serious worldwide health concerns in the twenty-first century and its prevalence has increased at an alarming rate. The prevalence of obesity in Thailand is one of the highest in Asia [1]. In 2011, the National Thai Food Consumption Survey examined the prevalence of overweight and obesity in a nationally representative sample of Thai children and adults [2]. Among children and adolescents aged three to eighteen years, the prevalence of overweight and obesity were 9.1% and 6.5%, respectively. Using the World Health Organization (WHO) standard for Asian populations, the combined prevalence of overweight and obesity in Thai adults aged nineteen years and over was 23.9%.

Overweight and obese children are likely to stay obese into adulthood. In addition, they are more likely to develop noncommunicable diseases such as non-alcoholic fatty liver disease, diabetes, and cardiovascular disease at a younger age. Alarmingly, obesity is characterized by a low-level systemic inflammation that might lead to a reduction in immune system responsiveness to vaccines [3,4,5,6]. In research involving hepatitis B virus (HBV) vaccine, several studies showed that obesity was a risk factor for vaccine non-responsiveness [7,8,9]. In contrast, results in studies involving killed hepatitis A virus (HAV) vaccine were inconclusive. Reuman et al. [10] and Van der Wielen et al. [11] argued that a higher vaccine response was associated with lower body mass index (BMI) and lower weight. Yet, Lim et al. [12] demonstrated that obesity did not significantly affect immunogenicity. Currently, there is no data focused on live HAV vaccines.

WHO recommends that any vaccination against HAV be integrated into the national immunization schedule for children aged ≥1 year [13,14]. Both inactivated and live attenuated HAV vaccines are highly immunogenic, generating long-lasting protection against HAV in children and adults. Presently, there is a trend to use the live HAV vaccine variant because it involves a single dose. In 2014, the Indian Academy of Pediatrics (IAP) updated their recommendations for live attenuated HAV vaccine in favor of the single dose variant as opposed to the previous two-dose schedule [15].

Because of the limited amount of immunogenicity data for live attenuated HAV vaccine in obese populations, the primary objective of our study was to evaluate the effects of overweight and obesity on the immune response to live attenuated HAV vaccine in children and young adults. Secondary objectives included identifying potential factors associated with postvaccination anti-HAV titers and assessing the reactogenicity of live attenuated HAV vaccine in non-obese and obese vaccine recipients.

## 2. Materials and Methods

### 2.1. Study Population

This was a prospective cohort study conducted between November 2019 and February 2020. All subjects were aged seven to twenty-five years and recruited from two primary schools, one secondary school, one university in Bangkok and the outpatient obesity clinic of King Chulalongkorn Memorial Hospital (KCMH). Written informed consent was obtained from each participant aged 18 years or older or from the parent of any participant younger than 18 years. Written informed assent was also obtained from each participant aged seven to eighteen years. Participants who had underlying immunodeficiency diseases, were previously immunized with an HAV vaccine, or had a seropositive (anti-HAV ≥ 20 mIU/mL) blood sample were excluded from study analysis.

#### Anthropometric Measurements

Standard, calibrated scales and stadiometers were used to determine height, weight, waist circumference, and body mass index (BMI = weight (kg)/height (m)^2^). Participants were classified as underweight, normal weight, overweight, obese, and severely obese according to their BMI using the WHO Child Growth Standards for participants aged seven to eighteen years and the WHO standards for Asian populations for participants aged nineteen years and older [16,17,18] (Supplement Table S1). Participants were then divided into two subgroups for study analysis, i.e., Group 1: Non-obese group, comprised of underweight or normal weight subjects; and Group 2: Obese group, comprised of overweight, obese or severely obese subjects. 

Cutoff points for waist circumference (WC), as defined by International Diabetes Federation criteria with adjustments for Asian populations [19,20,21], were used to determine truncal obesity in this study.

The study was conducted in accordance with ICMR guidelines for biomedical research on human subjects and the Declaration of Helsinki. This study was registered in the Thai Clinical Trials Registry (TCTR) (ID number TCTR20191230001).

### 2.2. Study Vaccine

The enrolled subjects were vaccinated (subcutaneous over the deltoid muscle of the upper arm) with 0.5 mL of MEVAC™-A. The MEVAC™-A (H_2_ strain, freeze dried, live) vaccine was developed by Zhejiang Pukang Biotechnology Co, Ltd., People’s Republic of China.

#### 2.2.1. Assessment of Immunogenicity and Serological Assay

Blood samples, measuring the levels of anti-HAV antibodies, were taken from each subject one day before the study vaccination and 8–9 weeks after the vaccination day. Based on earlier studies, an anti-HAV IgG titer of ≥20 mIU/mL was considered seroprotective [22].

Anti-HAV titer was determined by electrochemiluminescence immunoassay (ECLIA) on a cobas e 801 immunoassay analyzer according to the manufacturer’s instructions (Roche Diagnostics, Mannheim, Germany). Summary statistics for HAV-antibody concentrations from the immunoassay analyzer were used to calculate the geometric mean titers (GMTs).

#### 2.2.2. Assessment of Safety

Vaccine safety was assessed on the basis of observed or reported adverse events (AEs). Every vaccinated subject was observed closely at the study site for one hour postvaccination, with follow-up telephone calls using a standardized questionnaire at 3 days (72 h) after study vaccination to gauge local/systemic reactions. Information on any serious AEs, reported by the vaccinees through the end of the study, was also collected.

### 2.3. Statistical Analysis

Multiple linear regression (MLR) was used to assess the association between obesity and logarithm base 10 HAV-antibody titer and to identify any factors associated with the HAV-antibody titer. The minimum sample size was estimated using the “50 + 8 m” formula proposed by Tabachnick and Fidell [23], where “m” is the number of factors. Initially, five to six factors associated with HAV-antibody titer were expected following vaccination. As a result, the minimum number of subjects for the study was set at 98. Based on suggestion by Siddiqui [24], the sample size of 200 is fairly sufficient for multivariate analysis. Adjusting for a dropout rate of 15%, a total sample size of 230 subjects was targeted in order to have at least 200 subjects for the study analysis.

Statistical analysis was performed using STATA 15.1 (StataCorp, College Station, TX, USA). Categorical data were expressed as a number and percentage. Continuous data were given as the mean with standard deviation (SD), whereas GMT values were expressed as the mean with 95% confidence interval (95% CI). Comparisons between two groups were analyzed using the student t-test or Mann-Whitney U test for continuous data and the Chi-square or Fisher’s exact tests for categorical data. Associations between factors (e.g., obesity group, baseline titer, gender, age, truncal obesity, and acanthosis nigricans) were analyzed by simple and multiple linear regression using the backward stepwise elimination method. *p*-values less than 0.05 were considered to be statistically significant.

## 3. Results

### 3.1. Participants

**Figure 1:**
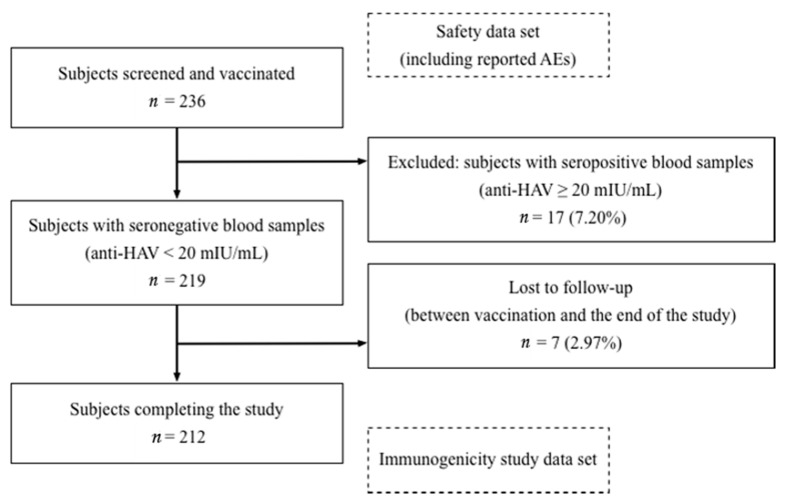
Disposition of subjects in the study.

Of the 236 subjects screened and given the study vaccination, 24 subjects (10.17%) were excluded from the study analysis either for having seropositive blood serum (*n* = 17, 7.20%) or for not following up between study vaccination and the end of the study (*n* = 7, 2.97%). The remaining 212 subjects were included in the immunogenicity analysis. For the safety analysis, all subjects injected with the study vaccine were included in the data set. The disposition of subjects is presented in Figure 1.

#### Baseline Demographic and Clinical Characteristics

From the group of 212 subjects who completed the study, 131 were female (61.79%) and 78 presented with truncal obesity (36.79%). The mean age ± SD of those who completed the study was 13.95 ± 3.90 years. A total of 117 subjects (55.19%) were allocated to the non-obese group while the remaining 95 subjects (44.81%) were allocated to the obese group. For the non-obese group, mean ± SD BMI and waist circumference values were 18.49 ± 2.87 kg/m^2^ and 65.16 ± 7.55 cm, respectively. For the obese group, mean ± SD BMI and waist circumference values were 28.30 ± 8.00 kg/m^2^ and 88.62 ± 18.07 cm, respectively.

There were no statistically significant differences in baseline anti-HAV titer and age between the non-obese and obese groups. However, a significantly higher proportion of women was observed in the non-obese group as opposed to the obese group (82 subjects (70.09%) versus 49 subjects (51.58%)). In the obese group, 33 subjects (34.74%) presented with acanthosis nigricans. Table 1 displays the baseline demographics and characteristics of the study subjects.

### 3.2. Postvaccination Immunogenicity

The time from vaccination to anti-HAV titer measurement, given as mean ± SD, was 8.47 ± 0.48 weeks (8.50 ± 0.46 weeks for the non-obese group and 8.43 ± 0.51 weeks for the obese group, *p* = 0.310). Overall postvaccination anti-HAV GMT (95% CI) was 446.11 (421.64, 472.01) mIU/mL. All non-obese and obese subjects (100%) were seroprotected after a single dose of live vaccine. The postvaccination anti-HAV GMTs (95% CI) for the non-obese and obese groups were 429.51 (401.97, 458.94) and 467.45 (424.47, 514.79) mIU/mL, respectively (*p* = 0.142) (Supplement Table S2).

Both simple regression and MLR analyses on the postvaccination log 10 anti-HAV results (Table 2) showed no significant differences in the anti-HAV titers between the non-obese and obese groups. In addition, simple regression and MLR analyses of the log 10 anti-HAV titer after vaccination showed no difference in the titers between children (≤18 years) and adults (>18 years). However, significantly higher antibody titer responses at 8–9 weeks after vaccination were found for female subjects (MLR *p*-value: 0.013) and subjects with truncal obesity (MLR *p*-value: 0.002).

Additional exploratory analyses to determine the effect of different influencing factors (e.g., baseline GMT, truncal obesity, sex, age group, grading of obesity (overweight, obesity, and severe obesity) and acanthosis nigricans) on the GMT values for anti-HAV in the obese group (*n* = 95) were performed by MLR analysis using the backward stepwise elimination method on log 10 anti-HAV values. The results show that the postvaccination anti-HAV titer was influenced by truncal obesity only (Table 3).

### 3.3. Safety and Adverse Events

In the 72-h postvaccination observation period, two out of 236 vaccinees did not respond to the postvaccination phone call. From the remaining 234 respondents, a total of 106 adverse events were reported. The most common adverse events were myalgia (reported by 15.38% of respondents or 36 vaccinees), headache (12.82%), fever (11.11%), swelling and pain at the injection site (4.27%), and abdominal discomfort (1.71%) (Table 4). Neither serious AEs nor requiring hospitalization were observed during the study. There were no differences in the rates of adverse events between the non-obese and obese groups.

## 4. Discussion

The continuing decline in hepatitis A infection prevalence induced by a rise in socioeconomic status, improvements in sanitary conditions, and the availability of a hepatitis A vaccine has been observed in many parts of the world including Thailand [25]. Results from a 2014 survey in Thailand reported an age-standardized hepatitis A seroprevalence rate of 48.6%, revealing a much lower value among younger age groups than corresponding studies from 1971–1972 (86.4%), 1976 (59.7%), and 1996 (64.5%) [26]. The present study corroborates this decreasing trend, finding a prevalence of hepatitis A infection in subjects aged seven to twenty-five years of only 7.2%, as determined by the number of subjects with an initial seropositive blood sample. This significant change in hepatitis A epidemiology has resulted in a rising number of symptomatic cases on account of the increase in average age at infection. Consequently, immunization against HAV is a useful tool to prevent symptomatic infections and outbreaks.

Zhang et al. [27] reported that a single dose of either inactivated HAV (Healive^®^) or live attenuated HAV (H_2_ strain) resulted in seroprotection for 70% of their subjects, with the inactivated vaccine having a higher immunogenic potential 24 months postvaccination. In our study, the seroprotection rate was 100% at eight to nine weeks postvaccination, which is in agreement with the two previous studies using live attenuated hepatitis A vaccine that reported seroprotection rates of 95.8% [28] and 99% [22] at six and eight weeks after vaccination, respectively. As of 2019, two live attenuated HAV vaccines are available but their use is presently limited to China, with scattered use in other countries such as India [29] and Thailand.

Individuals with fatty liver disease, a common complication of obesity, may develop chronic liver disease and ultimately cirrhosis. Such individuals carry a higher risk for developing severe liver impairment and fulminant hepatic failure, should a HAV infection occur. Therefore, vaccination against HAV is generally recommended in patients with chronic liver diseases. Obesity is considered a low-grade chronic inflammatory condition [30,31,32], placing obese individuals in a pro-inflammatory state that may interfere with immunogenicity to vaccine formulations. A review of influenza virus by Honce and Schultz-Cherry [33] notes how a baseline inflammatory state can negatively influence the induction of an adequate response to virus, resulting in longer infections, delayed viral clearance, and increased viral shedding. In addition, prior research has shown that obesity reduces the antibody response to a number of vaccines, including those for HBV, inactivated HAV, influenza, tetanus, and rabies [6]. Yet, there are studies showing that obesity is not a significant factor for poor seroprotection following immunization [12,34,35]. Our study also finds that obesity is not a risk factor for non-responsiveness to live attenuated hepatitis A vaccine. Obese participants did have a higher antibody response to live HAV vaccination than non-obese subjects but there was no statistical difference. Though our study lack data on complete blood count with differential along with inflammatory markers, such as high-sensitivity C-reactive protein and pro-inflammatory cytokines, the correlation between these markers and vaccine response should be further explored.

To date, there is limited data regarding the impact of obesity on the immune system response to live attenuated vaccines. Bollaerts et al. [36] examined the risk factors influencing vaccine effectiveness for the live attenuated zoster vaccine among 111,376 elderly subjects in England and found that zoster vaccine effectiveness was not altered by BMI. Through the use of diet-induced obese (DIO) mice, Estrada, Vazquez-Pagan, and Schultz-Cherry [37] reported that live attenuated influenza virus was successful in protecting obese hosts from severe disease through the action of elevated effector memory CD4 and CD8 T cells in mouse lung tissue. Although research on immune responses to live attenuated HAV vaccine in obese subjects is sparse, a relationship between obesity and inactivated HAV vaccine-induced immune responses has been reported by three groups, all showing variable results. Reuman et al. [10] showed that weight (*p* = 0.019) and BMI (*p* = 0.016) were significant negative predictors of seroresponse (anti-HAV titers ≥ 10 mIU/mL) when anti-HAV titers were measured in 100 participants in the United States (half of whom were younger than 40 years) four weeks after vaccination with alum-adjuvanted formalin-inactivated hepatitis A vaccine. Later, Van der Wielen et al. [11] measured anti-HAV levels seven months after vaccination, with the combined HAV/HBV vaccine (Twin-rix^®^), in 596 adult subjects from Europe (Germany, Belgium, and Czech Republic). The most consequential factor correlated with a significant decline in anti-HAV titers was an elevated BMI. Yet Lim et al. [12] found no difference in anti-HAV titers, measured eleven months after the primary dose of inactivated HAV vaccine, between healthy and obese individuals, indicating that obesity did not significantly affect seroconversion following hepatitis A vaccination in their subjects [451 medical school students, of which 98.7% were aged 17–22]. Similarly, Sheridan et al. [38] showed that obese participants had a vigorous initial antibody response to inactivated trivalent influenza vaccine. Nevertheless, when examining the level of antibody maintenance twelve months after immunization, higher BMI values were positively correlated with a four-fold or greater drop in influenza antibody titer. Along with this significant decline in influenza antibody levels, CD8+ T-cell responses were found to be defective in obese participants when compared to normal weight participants. Studies monitoring antibody maintenance levels for longer time durations following live attenuated hepatitis A vaccination may uncover other differences between non-obese and obese populations [39].

Although seroconversion rates in adults were similar between male and female populations [40], females consistently mounted higher anti-HAV antibody responses than males [1,41,42]. The present study also showed that females develop significantly higher immunogenicity than males. Although female gender was a borderline significant univariate predictor (*p* = 0.044), it became a significant factor associated with higher antibody response based on multivariate analysis (*p* = 0.013). A possible explanation lies in the observation that major sex steroid hormones promote opposing effects on the cells of the adaptive and innate immune systems, e.g., estradiol may play an enhancing role while testosterone may play a suppressing role [43]. As a result, immune responses to some vaccines will differ between male and female subjects [44]. For the influenza and hepatitis B vaccines, females exhibit a stronger humoral response. However, this is not the case for the pneumococcal polysaccharide vaccine, where males display a stronger response. We are unaware of any study that differentiates between the male and female responses to live attenuated HAV vaccination.

Currently, there are limited data regarding how waist circumference, truncal obesity, or metabolic syndrome may be linked to vaccine efficacy. Our study revealed that truncal obesity subjects mounted a significantly higher anti-HAV response than the balance of the study population. Truncal obesity involves an increase in lipid storage within adipocytes, as evidenced by a buildup of adipose tissue in the abdominal region. Because adipose tissue has an inherent endocrine function as well as a lipid storage function, it can exhibit varying pathologies according to its classification as either visceral or peripheral/truncal adipose tissue. Whereas visceral adipose tissue is associated with conditions such as dyslipidemia, cardiovascular disease, and type-2 diabetes, peripheral adipose tissue protects against dysfunction by collecting excess lipid that would otherwise accumulate in other tissues [45]. As adipocytes engage in endocrine signaling, they release a variety of adipokines including leptin, which is capable of regulating innate and adaptive immune responses. In vitro and in vivo studies have revealed that leptin is a crucial factor for T-cell proliferation and differentiation [46]. Moreover, adipocytes express functional pattern recognition receptors and can subsequently respond to viral antigens, strengthening the link between adipose tissues and the immune system signaling network [47]. Because females have a greater amount of adipose tissue than males (20–30% and 10–20% of total body weight in females and males, respectively) [48], the properties of adipocytes and their greater presence in females may explain the elevated immunogenicity to HAV vaccine among females and truncal obesity subjects in the present study. Stoddart et al. [49] studied in 2205 participants aged 5–40 years (454 were 5–19 years of age) and reported that acanthosis nigricans was independently associated with hyperinsulinemia. A study in 74 obese children revealed that individuals with acanthosis nigricans had significantly greater fasting insulin level and homeostatic model assessment insulin resistance (HOMA-IR) score [50]. These data support the role of acanthosis nigricans as a potential early indicator of high risk for diabetes. Our study demonstrated the comparable immunogenicity between obese subjects with and without acanthosis nigricans.

Prior investigations indicate that live attenuated HAV vaccine is safe and well tolerated [13,20,22,51,52], with no reports of any serious AEs related to the vaccine. The most commonly observed AEs were fever and pain, along with redness and swelling at the injection site, all of which resolve themselves within a few hours or days [10]. In the present study, there was no difference between the non-obese and obese participants in terms of observed AEs during the safety follow-up duration of 8–9 weeks, all of which were mild.

Age and gender matching of controls were not performed for this study. To expand our findings of excellent immunogenicity for a single HAV vaccine dose, both a long-term follow-up study to determine the persistence of vaccine protective efficacy and a short-term study to evaluate the potential protective efficacy during imminent viral outbreaks or travel preparations are necessary.

## 5. Conclusions

A single vaccination using the live attenuated hepatitis A vaccine is safe and highly immunogenic in both underweight/normal weight and overweight/obese subjects during the short-term follow-up. Truncal obesity and female gender are factors associated with better immune response, whereas there are no differences in anti-HAV titers between non-obese versus obese groups or between children versus young adults.

## Figures and Tables

**Table 1:** Baseline characteristics by nutritional status groups.

		Total (*n* = 212)	Non-Obese Group (*n* = 117)	Obese Group (*n* = 95)	*p*
Gender, *n* (%)	Male	81 (38.21)	35 (29.91)	46 (48.42)	0.006 ^1,^*
	Female	131 (61.79)	82 (70.09)	49 (51.58)	
Age (years) [mean (SD)]		13.95 (3.90)	14.20 (3.47)	13.63 (4.37)	0.288 ^2^
Age group ^3^, *n* (%)	Children	172 (81.13)	95 (81.20)	77 (81.05)	0.979 ^1^
	Adults	40 (18.87)	22 (18.80)	18 (18.95)	
Truncal obesity, *n* (%)	No	134 (63.21)	113 (96.58)	21 (22.11)	<0.001 ^2,^*
	Yes	78 (36.79)	4 (3.42)	74 (77.89)	
Anti-HAV titers, (mlU/mL) [GMT (95% CI)]		2.60 (2.27, 2.97)	2.63 (2.19, 3.15)	2.56 (2.09, 3.13)	0.851 ^2^

^1^ Chi-square test, ^2^ T-test, ^3^ children; age ≤ 18 years and adult; age > 18 years; * Statistically significant with *p*-value < 0.05; The non-obese group includes normal and underweight subjects while the obese group includes overweight, obese, and severely obese subjects.

**Table 2:** Association between baseline characteristics and postvaccination anti-hepatitis A virus (HAV) titers.

	*n*	Postvaccination Anti-HAV Titers, GMT (95% CI)	Univariate *p* ^1^	Multivariate *p* ^2^
Baseline anti-HAV titers	212	2.60 (2.27, 2.97)	0.416	
Nutritional status ^3^				
Non-obese group	117	429.51 (401.97, 458.94)	Ref	
Obese group	95	467.45 (424.47, 514.79)	0.142	
Age groups				
Adults	40	477.78 (411.03, 555.37)	Ref	
Children	172	439.06 (413.22, 466.52)	0.249	
Truncal obesity				
No	134	420.78 (395.28, 447.92)	Ref	Ref
Yes	78	493.26 (442.86, 549.39)	0.007 *	0.002 *
Gender				
Male	81	414.58 (372.97, 460.83)	Ref	Ref
Female	131	466.81 (438.02, 497.49)	0.044 *	0.013 *

^1^ Simple linear regression analysis of log 10 anti-HAV titers; ^2^ multiple linear regression (MLR) analysis with backward stepwise elimination of log 10 anti-HAV titers; ^3^ the non-obese group includes normal and underweight subjects while the obese group includes overweight, obese, and severely obese subjects. * Statistically significant with *p*-value < 0.05; Abbreviations: Ref, reference group. Final MLR model containing 2 variables was “Log 10 anti-HAV titers = 2.52 + 0.078 (if truncal obesity) + 0.062 (if female).” Baseline log 10 anti-HAV titers, nutritional status (non-obese or obese groups), and age group were removed from the full model with backward stepwise elimination with *p*-value < 0.05.

**Table 3:** Association between baseline characteristics and postvaccination anti-HAV titers in obese group.

	*n*	Postvaccination Anti-HAV Titers, GMT (95% CI)	Univariate *p* ^1^	Multivariate *p* ^2^
Baseline anti-HAV titers	95	2.56 (2.09, 3.13)	0.050	
Obesity levels				
Overweight	34	436.69 (380.27, 501.45)	Ref	
Obesity	31	474.37 (410.64, 547.99)	0.485	
Severe obesity	30	497.38 (395.36, 625.72)	0.277	
Age groups				
Adults	18	462.59 (355.63, 601.72)	Ref	
Children	77	468.60 (421.82, 520.56)	0.919	
Truncal obesity				
No	21	371.07 (314.18, 438.25)	Ref	Ref
Yes	74	499.11 (446.37, 558.08)	0.011 *	0.011 *
Gender				
Male	46	449.93 (381.09, 531.21)	Ref	
Female	49	484.53 (434.63, 540.15)	0.449	
Acanthosis nigricans				
No	62	442.11 (400.04, 488.61)	Ref	
Yes	33	519.06 (421.40, 639.36)	0.116	

^1^ Simple linear regression analysis of log 10 anti-HAV titers; ^2^ multiple linear regression analysis with backward stepwise elimination of log 10 anti-HAV titers; * Statistically significant with *p*-value < 0.05; Abbreviations: Ref, reference group; Final MLR model was “Log 10 anti-HAV titers = 2.57 + 0.13 (if truncal obesity).” Age group, gender, levels of obesity, acanthosis nigricans, and baseline log 10 anti-HAV titer were removed from the full model with backward stepwise elimination with *p*-value < 0.05.

**Table 4:** List of adverse events observed within 72 h postvaccination.

Adverse Event	Number of Vaccinees			*p*
	Total (*n* = 234) *	Non-Obese (*n* = 126)	Obese (*n* = 108)	
No complication, *n* (%)	165 (70.51)	85 (67.46)	80 (74.07)	0.269
Fever, *n* (%)	26 (11.11)	15 (11.90)	11 (10.19)	0.677
Headache, *n* (%)	30 (12.82)	17 (13.49)	13 (12.04)	0.740
Myalgia, *n* (%)	36 (15.38)	22 (17.46)	14 (12.96)	0.342
Abdominal discomfort, *n* (%)	4 (1.71)	2 (1.59)	2 (1.85)	1.000
Swelling and pain at the injection site, *n* (%)	10 (4.27)	5 (3.97)	5 (4.63)	1.000

* 2 out of 236 vaccinees did not respond to the 72-hour postvaccination phone call. *p*-value from Chi-square or Fisher’s exact as appropriate.

## Data Availability

Not applicable.

## Supplementary Materials

The following are available online at https://www.mdpi.com/2076-393X/9/2/130/s1. Table S1: Classification of nutritional status by body mass index (BMI) in children and Asian adults. Table S2: GMT anti-HAV titers in different WHO BMI classifications.
