# Supplementary material for: Impact of Obesity and Being Overweight on the Immunogenicity to Live Attenuated Hepatitis A Vaccine in Children and Young Adults
Source: Vaccines (Basel). 2021 Feb 6;9(2):130. doi: 10.3390/vaccines9020130 (PMC7915133; doi:10.3390/vaccines9020130)
Supplement: Supplementary file 1 [file vaccines-09-00130-s001.pdf]

Table S1. Classification of nutritional status by body mass index (BMI) in children and Asian adults.

Classification	Age 7-19 years BMI Z-score	Adult (aged > 19 years) BMI (kg/m^2)	Categorized in this study
Underweight	< -2SD	< 18.5	Non-obese group
Normal weight	-2SD to +1SD	18.5-22.9	
Overweight	> +1SD to ≤ +2SD	23-24.9	Obese group
Obesity	> +2SD to ≤ +3SD	25-29.9	
Severe obesity	> +3SD	≥ 30	

Table S2. GMT anti-HAV titers in different WHO BMI classifications.

	**Underweight (n=6)**	**Normal weight (n=111)**	**Overweight (n=34)**	**Obesity (n=31)**	**Severe obesity (n=30)**	***p***
Anti-HAV titers (mlU/mL) [GMT (95% CI)]	451.10 (374.76, 543.0)	428.38 (399.61, 459.22)	436.69 (380.27, 501.45)	474.37 (410.64, 547.99)	497.38 (395.36, 625.72)	0.427
